# Investigation on the Carbonation Behavior of Alkali-Activated Pastes Served under Windy Environments

**DOI:** 10.3390/ma16020825

**Published:** 2023-01-14

**Authors:** Dong Cui, Lingshu Shen, Yidong Shen, Guantong Han, Xiaoying Xie, Qianfei Cao, Jing Wang, Hao Wei, Qiannan Wang, Keren Zheng

**Affiliations:** 1 Department of Civil Engineering, School of Science, Nanjing University of Science and Technology, Nanjing 210094, China; 2 School of Civil Engineering and Architecture, Zhejiang University of Science & Technology, Hangzhou 310023, China; 3 Department of Civil Engineering, Central South University, Changsha 410075, China

**Keywords:** alkali-activated slag, carbonation, wind environment, cementitious materials, durability

## Abstract

Most reinforced concrete structures serve under windy environments, and the carbonation resistance under that circumstance exhibits significant difference from that under the steady (no wind) environment. In this study, a windy environment was simulated using one self-developed wind tunnel, and alkali-activated slag/fly ash paste specimens were adopted for the carbonation under variant windy environments. Meanwhile, to reveal the effect of inner humidity on the carbonation, sliced alkali-activated materials (AAM) were mass-balanced first to variant humidity, and were then carbonated under a 2.5 m/s windy environment. With the assistance of computed tomography (CT), the structure of AAM at variant carbonation ages was rendered. The experimental result showed that wind is capable of promoting the exchange of moisture between the sample inside and the outer atmosphere, leading to faster carbonation as compared to that under no wind environment. When preconditioned to lower inner humidity, the carbonation rate of AAM was faster because the larger gaseous space benefited the intrusion of both CO_2_ and moisture. Furthermore, when preconditioned to lower humidity, the cracking extent of AAM was severer, which also contributed to the faster carbonation. Moreover, compared with ordinary Portland cement (OPC), the carbonation front on each instant 1D gray-scale value profile was broader, which suggested that the carbonation progress of AAM under windy environments was no longer controlled solely by diffusion. In addition, the gray-scale value on instant 1D profile fluctuated drastically, which verified cracking in AAM carbonated under windy environments. The current work not only deepens the understanding of the carbonation mechanism in-site (mostly under windy environments), but also helps to develop more environment-friendly construction material, with better durability performance.

## 1. Introduction

At present, China is still going through the rapid construction of infrastructures. National projects, such as the construction of the Hong Kong-Zhuhai-Macao Bridge [1], the Beijing New Airport [2], and the Sichuan-Tibet Railway [3], all injected new impetus to the national economy growth. However, since China is located in the Northern Hemisphere, the whole country suffers from a northeast trade wind all year around [4]. In addition, with complex terrains, the inland mountains and the coastal areas of China also tend to be vulnerable towards the valley wind, the land-sea wind, and the monsoon [5,6]. Serving under such a complicated windy environment, the concrete surface will gradually absorb the CO_2_ in the air. The absorption reduces the alkalinity of the concrete, accelerates the corrosion of the rebar buried inside concrete, and eventually leads to failure of the whole construction [7,8,9]. Such a carbonation issue under windy environments has been reported worldwide. Take the RC power in Baku, Russia as an example: after years of serving under a windy environment, the carbonation depths of both the windward and leeward faces were 1.5 to 2 times larger than that on the remained faces [10]. Another example was offered by Qu et al. [11], where the windy environment was proven detrimental to the durability of concrete beams. Therefore, it is important to understand the carbonation mechanism of reinforced concretes serving under windy environments, as it promises an accurate durability design of the reinforced concretes serving in-site (mostly under windy environments). 

Unfortunately, the carbonation mechanism under a windy environment is very complex. Aside from the wind pressure which promotes the invasion of CO_2_, the evolved inner humidity under variant windy environments also influences the carbonation process. For that reason, the carbonation test performed under a windy environment remains limited at the current stage. Li et al. [12] simulated an aeolian washout environment using high-speed wind carrying sands, and a carbonation test was conducted under that simulated condition. The experimental result verified that both the lash by the washout and the deformation by carbonation deteriorated the microstructure of concrete structures. Even though the work is innovative, the aeolian washout was not a common windy environment, so the revealed mechanism was more applicable for harsh natural environments. Therefore, in order to reveal the carbonation mechanism in-site, carbonation under a milder windy environment should be carried out next. 

On the other hand, for the purpose of achieving the carbon neutralization, a potential substitute of the Portland cement, such as the alkali-activated materials (AAM), is gaining increased concentration in recent years [13,14]. Compared with the ordinary Portland cement (OPC), the strength of AAM develops more rapidly [15,16], and the resistance against the invasion of chloride [17,18] and sulfate ion [19,20] are better. Even so, the use of AAM has to be made with caution. Primarily, due to the higher risk of cracking [21,22,23], the carbonation resistance of concrete structures was worse than OPC, as the cracks in AAM can serve as the “fast path” for CO_2_ invasion [24,25,26,27]. In addition, the calcium content in AAM is lower than that in OPC, which also contributes to the weaker carbonation resistance of AAM, as less of a carbonation-buffering agent (e.g., calcium hydroxide) was available [28]. To massively promote the use of AAM in construction projects, the investigation on AAM carbonation in-site (mostly under windy environments) should be carried out in the future. 

In the present study, a windy environment was simulated through one self-developed wind tunnel, and alkali-activated slag/fly ash paste specimens were carbonated under the simulated windy environment. To reveal the effect of inner humidity on the carbonation of AAM under a windy environment, sliced AAM pastes were mass-balanced first, and were then carbonated under the windy environment. With the help of computed tomography (CT), the carbonated area and the crack morphology at variant carbonation ages were both obtained, and the effect of wind on the carbonation of AAM was systematically investigated. The current work not only deepens the understanding of the carbonation mechanism in-site (mostly under windy environment), but also helps to develop more environment-friendly construction material, with better durability performance. 

## 2. Materials and Methods

### 2.1. Raw Materials

Blast furnace slag (SL) and fly ash (FA) were used in the present study. The fly ash with a density of 3000 kg/m^3^ was produced by an iron plant in Inner Mongolia, China. The slag with a density of 2800 kg/m^3^ was produced by Rongda Company in Jiangsu Province, China. The specific surface of raw materials was calibrated by a surface area analyzer manufactured from Xinmaoluye Company in Hebei Province, China. The chemical composition of both materials were calculated based on one X-ray fluorescence (XRF) analyzer manufactured by Allalin Company in Switzerland. The raw materials were ground roughly first. Next, 20 g of powder and 2 g of stearic acid were put in a vibrating mill, and were ground for another 1 min. After that, powders were pressed under 30 MPa pressure to form the sample for XRF analysis. The obtained chemical compositions are listed in Table 1.

Sodium hydroxide (NaOH) and sodium silicate (Na_2_O·nSiO_2_) were mixed to formulate the alkali exciter. Flaky NaOH solid with a purity of 96% was produced by Kermel Company in Tianjin City, China, and Na_2_O·nSiO_2_, with a modulus of 1.03 was produced by Hengxi Company in Tianjin, China. The mass fractions of SiO_2_ and Na_2_O in Na_2_O·nSiO_2_ were 30.5% and 29.6%, separately, and the mass ratio of NaOH, Na_2_O·nSiO_2,_ and water was designed as 1:5:30. The modulus of the formulated alkali activator was 1.5, and the alkali content was about 4.5%. 

### 2.2. Sample Preparation

In order to systematically understand the carbonation behavior of AAM under windy environments, aside from the AAM made of pure slag (denoted as “SL100”), alkali-activated pastes, with 25% (denoted as “SL75”) and 50% (denoted as “SL50”) by mass of slag replaced by the fly ash, were also prepared in present study. The water to binder ratio for all AAM was 0.45. To guarantee sufficient dissolution of Na_2_O·nSiO_2_, alkali exciter was dissolved 12 h ahead of the formulation. During the sample casting, slag and fly ash powders were dry-mixed first for 2 min in a mixer, and the alkali exciter was poured next in the same mixer. The raw materials and the exciter was slow-mixed, paused, and quick-mixed subsequently for 120 s, 15 s, and 120 s. After that, pastes were set directly in molds, and were taken for an additional 3-month curing under sealed condition. 

Two shapes of specimens were prepared. Cylindrical specimens (Φ 20 mm × 100 mm) were cast and cured in PVC molds, and the conjunction between the PVC mold and the AAM matrix was sealed carefully by epoxy resin before carbonation. Sliced specimens (40 mm × 40 mm × 5 mm) were mass-balanced first to a designed humidity level (30%, 50%, or 70%), and five side faces of each slice were sealed next by epoxy resin, leaving one (40 mm × 5 mm) for carbonation under the windy environment. 

### 2.3. Carbonation Tests

**Figure 1:**
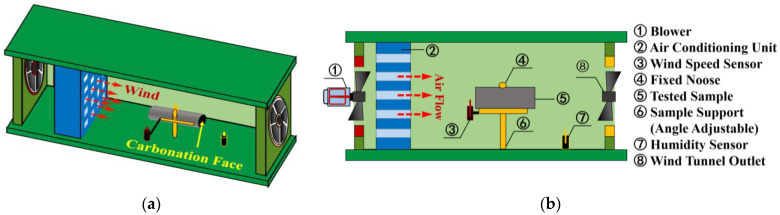
(**a**) 3D and (**b**) 2D Schematic of the Self-Developed Wind Tunnel.

A carbonation test was conducted in a carbonation chamber. No special preconditioning (thermal drying or mass balancing, aiming to remove the excessive moisture) was necessary in the present study, as a sealed curing regime was adopted. Mass balancing was performed on sliced samples, aiming at balancing the inner humidity before the carbonation. The climate inside the carbonation chamber was: 20 ± 2 °C, 60 ± 5% relative humidity (RH), and 1 ± 0.2% CO_2_ concentration. A self-developed wind tunnel was set in the carbonation chamber to simulate the windy environment (see Figure 1). The wind was generated by a blower fixed at the entrance of the tunnel, and an air conditioner was used to regulate the wind direction. Through switching the tap of the blower, the wind speed near the carbonation face could be adjusted to approximately 2.5 m/s and 5.5 m/s, separately. Each specimen was tied to a noose, and its leeward side was taken for carbonation. Note here that leeward face was selected in the present study, so the effect of wind pressure on AAM carbonation was minimized; in other words, the main parameter that affected the AAM carbonation was the evolved inner humidity during carbonation under variant windy environments [29]. 

The whole carbonation test lasted for several months, and it was almost impossible to have a blow incessantly work for that long period. Therefore, the blower worked ceaselessly for 16 h, and was at rest during the remaining 8 h every day. Furthermore, all experiments were carried out in the wind tunnel, if the air in the tunnel was not refreshed periodically, the CO_2_ concentration would gradually reduce during carbonation. Therefore, even for the 0 m/s windy environment, the blower worked 2 min every 2 h to update the climate in the wind tunnel. The specimens were taken, respectively, for analysis at variant carbonation ages. For convenience, in the following, the steady (wind speed: 0 m/s wind), weak (wind speed: 2.5 m/s), and normal windy (wind speed: 5.5 m/s) environments were denoted as group “S”, “W”, and “N”, respectively. 

### 2.4. Testing Methods

#### 2.4.1. X-ray Computed Tomography

X-ray computed tomography (CT) was used widely for the investigation of carbonation. Its principle has been elaborated before [30,31]. The CT manufactured from YXLON Company in Germany was used, and its effective resolution was 89 μm. Furthermore, working voltage of 195 KeV and working current of 0.3 mA were adopted, and the X-ray was filtered by a 0.5 mm aluminum sheet ahead of scanning. Moreover, to achieve images of higher signal-to-noise ratio, the time used for each projection was 5 s. 

The raw CT data was displayed as a series of continuous digital images, and self-developed MATLAB codes were used in the present study to rotate the CT data in space, so as to precisely render the cross section and the axial plane of each tested specimen. 

#### 2.4.2. Phenolphthalein Spray

Phenolphthalein spray was applied in the present study to verify the carbonated zones read by CT. The axial plane of the partly carbonated specimen was exposed carefully by a diamond saw, and the phenolphthalein indicator was sprayed on the exposed area. The carbonated zone after-spray remains colorless, while the non-carbonated zone shifts to pink fuchsia through a chromatic aberration. 

#### 2.4.3. X-ray Diffraction

A Bruker D8 Advance XRD with a silver X-ray tube was used in this study to understand the change of phase composition during the carbonation of AAM. Specimens were cut separately from the carbonated and non-carbonated area of AAM, and were pulverized and sieved before XRD analysis. The step size was 0.02°, and each specimen was scanned from 5° to 70° 2θ. The Bruker Eva software and the IDCC PDF-2 database were combined for the phase identification.

#### 2.4.4. Titrimetric Method after Combustion

The titrimetric method after combustion was used in the present study to analyze the carbon content. The samples were pulverized and sieved at 74 μm. After that, the powder was burned in a tubular furnace to 1200 °C under oxygen atmosphere. The CO_2_. generated along the process was absorbed by the ethanol-ethanolamine aborption solution, and the carbon content was then determined by titration, with thymolphthalein as the indicator and potassium ethoxide as the standard solution. 

#### 2.4.5. Weighing Method

A high-precision analytical balance manufactured from Xingyun Factory in Suzhou City, Jiangsu Province, China was used to measure the mass of partly carbonated samples at variant carbonation ages. The mass variation resulted from both CO_2_ binding and moisture migration [32], so the information could be used to evaluate both the carbonation extent and the inner humidity at a given carbonation age. The measuring accuracy of the balances was 0.1 mg, and the measuring up-limit was 500 g. Weighing tests were restricted to sliced samples in the present study, as its accuracy was seriously disturbed by the occasional peeling off during carbonation of cylindrical specimens. 

## 3. Results

### 3.1. Raw CT Data of Partly Carbonated AAM

**Figure 2:**
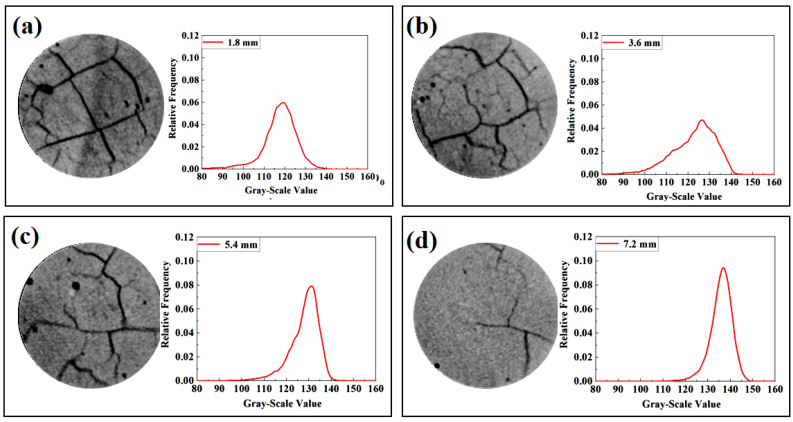
Typical cross sections rendered from (**a**) 1.8 mm, (**b**) 3.6 mm, (**c**) 5.4 mm and (**d**) 7.2 mm beneath the carbonation face of a SL50 cylindrical sample.

Figure 2 shows the typical digital images rendered from the cross sections of SL75. The wind speed was 2.5 m/s, and the CT scan was performed at 28 d of carbonation. From Figure 2a–d, the images were related to the cross section 1.8 mm, 3.6 mm, 5.4 mm, and 7.2 mm, respectively, beneath the carbonation face. As shown, honeycomb-shaped crack networks pervaded the cross section of the carbonated areas, but the network gradually vanished at deeper (non-carbonated) areas. The generation of cracks in the carbonated area is within our expectation since the hydration product changed from C-S-H in ordinary Portland cement (OPC) to C-(A)-S-H/ N-S-H in AAM. The autogenous shrinkage, drying shrinkage, and the carbonation shrinkage in SL75 were all orders of magnitude larger than that of OPC [21,22,23], and that leaded to severe cracking. Moreover, the cracks generated could serve as a “detour” to benefit the invasion of CO_2_, which also promoted the carbonation. Therefore, it is essential to enhance the cracking resistance of AAM, especially under windy environments. 

To quantitatively exhibit the carbonation result obtained by CT, histograms of the gray-scale values of each cross section were drawn, and the result is shown as well in Figure 2. The distribution of gray-scale value was broader in the carbonated area, but narrower in the deeper non-carbonated area. The result highlighted again the effect of cracking, which significantly diminished the homogeneity of AAM. In addition, it was interesting to report an unusual reduction of the gray-scale value within the carbonated area, as the most probable gray-scale value decreased from approximately 135 to approximately 120 moving from non-carbonated area to the carbonated one. Several reasons can be given here to explain the anomalous decrease of gray-scale value after carbonation. First, the gray-scale value of the carbonated area was “pulled down” by the crack network, as it acquired a relatively lower gray-scale value. Secondly, unlike C-S-H formed during cement hydration, the structure of alkali-activated products (e.g., C-(A)-S-H, N-S-H) was less stable, and a larger amount of water would release during its carbonation [14,25,28]. If the increase of gray-scale vale by CO_2_ binding was counterbalanced by the moisture loss, a decrease of the gray-scale value would then be inevitable. 

To be strict, several other parameters, such as the X-ray energy level used for the CT scan, and the effective resolution [33,34], could also cause the reduction of the gray-scale value in the carbonated area. The parameters worked together to reduce the gray-scale value of the carbonated area, and that could be used as a clue to trace the carbonation in AAM. 

### 3.2. Axial Plane of Carbonated AAM Exposed to Variant Windy Environment 

**Figure 3:**
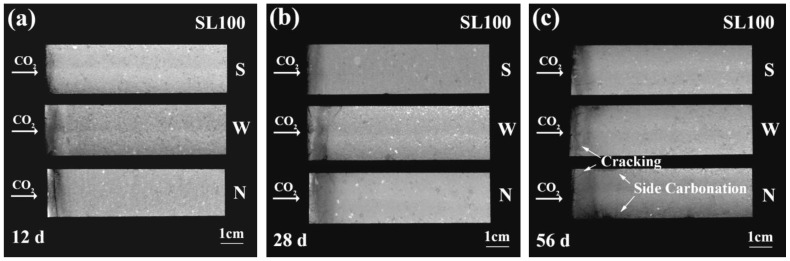
Rendered axial planes of SL100 at (**a**) 12 d, (**b**) 28 d, and (**c**) 56 d of carbonation. “S”, “W”, and “N” stand for steady, weak windy, and normal windy environments.

The cross section only offered the carbonation extent from a certain depth, and to achieve a full image on the carbonation extent of AAM under a windy environment, the axial plane (passing through the axis of each cylindrical specimen) of partly carbonated AAM was more suitable. The axial planes were attained through rotation of the raw CT data in space. Figure 3a–c show the axial planes of SL100 after 12 d, 28 d, 56 d of carbonation, respectively. To reveal the effect of wind velocity, each AAS was exposed, respectively, to three types of windy environments (wind speed of 0 m/s, 2.5 m/s, and 5.5 m/s, respectively). The carbonated area of SL100 was darker than that of the non-carbonated one, which agreed with Figure 2. The necessity to conduct carbonation tests under a windy environment was highlighted, as the wind of higher velocity was revealed to impulse a more significant impact on AAS carbonation throughout the testing period. Furthermore, note here that the leeward face, instead of the windward face, was adopted for carbonation, thus the faster carbonation was not caused by the wind pressure—quite the opposite, considering that the exchange of moisture was promoted by faster airflow, and thus the evolved inner humidity was referred to as the explanation for the faster carbonation. 

With closer observation, both the cracking resistance and the carbonation resistance of SL100 were promising because no clear sign of side carbonation emerged until 56 d of carbonation, even under a 5.5 m/s windy environment [see Figure 3c]. For side carbonation, it describes the anomalous carbonation through the leaked side faces of the tested specimen [35,36]. In fact, side carbonation was almost unavoidable, as preconditioning was essential in the laboratory: the saturation degree of the specimen was extremely high right after curing, as most specimens in the laboratory were either standard-cured or water-cured, and carbonation could not be carried out immediately due to lack of gaseous space for CO_2_ diffusion. For that reason, specimens must be thermal-dried or mass-balanced before carbonation, so as to achieve an intermediate humidity level (around 40% to 60%). However, preconditioning also introduced damages beneath the surface of side face, which could not be completely sealed by epoxy resin. Later, CO_2_ was inclined to diffuse through the cracks beneath the surface of side faces, leading to side carbonation [35]. The side carbonation was to a large extent avoided in the present study because all specimens were cured in a sealed condition, and all specimens were not demolded throughout the carbonation tests. Nonetheless, the sign of side carbonation still showed up at 56 d of carbonation, which suggested the damage (debonding) between the PVC mold and the specimen, most probably generated by the drying or carbonation shrinkage accumulated during carbonation. 

Despite the limited resolution of CT, cracks in AAM were still traceable. As shown in Figure 3c, cracks perpendicular to the carbonation face were observable in SL100 under all windy environments. Even so, the acceleration of carbonation by cracking seemed insignificant in SL100, as a regular-shaped carbonation front (without the presence of carbonation cusp) was universally observed. 

**Figure 4:**
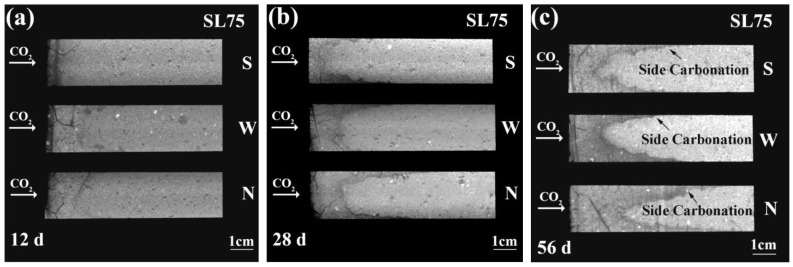
Rendered axial planes of SL75 at (**a**) 12 d, (**b**) 28 d, and (**c**) 56 d of carbonation. “S”, “W”, and “N” stand for steady, weak windy, and normal windy environments.

Figure 4a–c present the axial planes of SL75 at 12 d, 28 d, and 56 d of carbonation, respectively. The evolving pattern of carbonation appeared similar in SL100 and SL75, but the carbonation speed of SL75 was faster. Compared with SL100, the carbonation resistance of SL75 was weaker, and that complied with the former literature [37,38]. Two reasons are given here to explain the reduced carbonation resistance after the blending of fly ash. Primarily, the alkali-activated products gradually shifted from C-(A)-S-H to N-A-S-H after the incorporation of fly ash because the calcium content in type F fly ash was lower (see Table 1). The amount of calcium-bearing materials was reduced by the blending, and the carbonation resistance of SL75 was thus lower [38]. In addition, compared with the C-S-H formed during cement hydration, the calcium to silica (aluminum) ratio in C-(A)-S-H (formed by pozzolanic reaction) was lower, thus the microstructure after carbonation was coarser [39]. However, due to a “pore clogging” effect, the microstructure of concrete structures was densified after OPC carbonation. The coarsened microstructure also benefited the invasion of CO_2_, leading to faster carbonation. 

The effect of wind on the carbonation of SL75 was consistent with SL100. The carbonation speed increased gradually when facing an environment with faster wind. In addition, the side carbonation in SL75 was visited. The side carbonation was almost non-detectable at an early carbonation age [see Figure 4a], but emerged at 28 d of carbonation (see the concave meniscus in Figure 4b,c). Similar with the deduction given in SL100, the occurrence of side carbonation pointed to a leakage of the interface between the matrix and the PVC mold; although the current sealing strategy to a large extent avoided the damage beneath the surface of the side face, the interface between the SL75 and the PVC mold still tended to be more vulnerable as compared to that of SL75 matrix. Therefore, during carbonation, the shrinkage-incurred stress was preferable to release in the interface, causing the debonding of the interface. Later, CO_2_ was inclined to diffuse through the damaged interlayer, forming side carbonation. 

**Figure 5:**
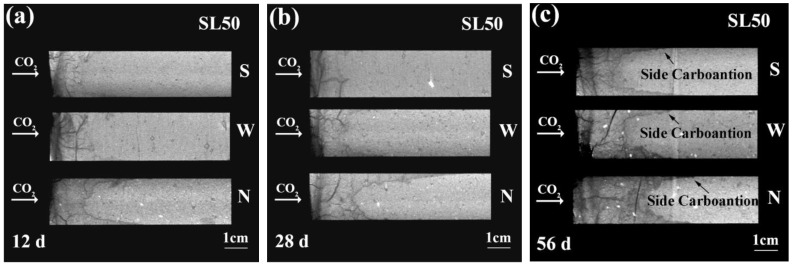
Rendered axial planes of SL50 at (**a**) 12 d, (**b**) 28 d and (**c**) 56 d of carbonation. “S”, “W”, and “N” stand for steady, weak windy, and normal windy environments.

Inheriting similar strategy, the axial planes of SL50 were generated, as shown in Figure 5. Compared with SL100 and SL75, the carbonation resistance of SL50 appeared the weakest, which was reasonable considering its extremely low calcium content [19]. Meanwhile, serious cracking was seen in Figure 5, which suggested the high shrinkage of SL50. In general, the cracking and carbonation posed positive feedback to each other: shrinkage was accumulated in AAM during carbonation, which lead to the generation/propagation of cracks; as a return, the generated/propagated cracks benefited the diffusion of CO_2_, and that lead to faster carbonation [40]. 

When exposing to an environment of faster wind, the promotion by the wind on carbonation was more evident. Even so, comparing SL75 and SL50, it is interesting to figure out that the lead of side carbonation was less significant in SL50. A case in point was the carbonation at 56 d. Even though the extent of side carbonation was similar for SL75 and SL50, the average carbonation depth of SL50 was obviously larger. The phenomenon could also be revealed from the less concave meniscus in Figure 5, and it reflected the cracking resistance of SL75 and SL50; as described earlier, shrinkage-induced cracking was the “culprit” behind the side carbonation of AAM in this study. Compared with SL50, the cracking resistance of SL75 was higher, and it was preferential for the shrinkage-incurred stress to be released within the interface between the AAM matrix and the PVC mold. For SL50, since its cracking resistance was weaker, the cracking probability of the matrix and the interface was similar, and that lead to less significant side carbonation. 

### 3.3. Validation of the Carbonation Result 

**Figure 6:**
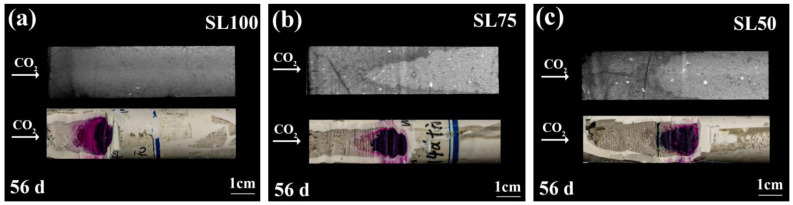
Rendered and exposed axial planes (after spray of phenolphthalein) of (**a**) SL100, (**b**) SL75, and (**c**) SL50. All specimens were exposed to a 5.5 m/s windy environment.

To validate the reliability of the carbonated areas revealed by CT, phenolphthalein spray was applied. The inner areas of SL100, SL75, and SL50 (subjected to a 5.5 m/s windy environment) were exposed by a diamond saw, and the axial planes after phenolphthalein spray are shown, respectively, in Figure 6. Note that the carbonated AAM was very fragile, thus all of the cutting process was conducted very carefully. As shown, the carbonated zone read from phenolphthalein agrees with the read from CT, verifying that the gray-scale value can be used to trace the carbonation in AAM. Better yet, it was noticed that the carbonation front could be clearly revealed by CT, while for specimens sprayed with phenolphthalein, the carbonation front was somehow vaguer. 

**Figure 7:**
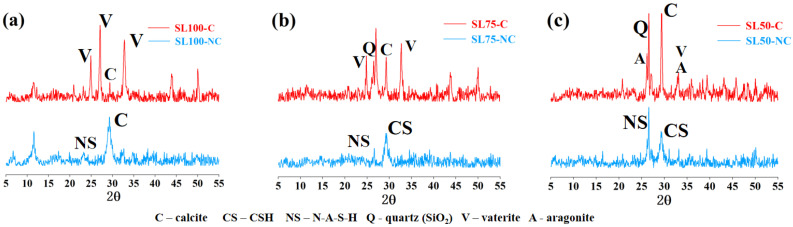
XRD diffractograms of non-carbonated and carbonated: (**a**) SL100, (**b**) SL75 and, (**c**) SL50 specimens. C: carbonated; NC: non-carbonated.

In order to verify the ongoing carbonation in AAM, the titrimetric method was used to measure the total calcium content. Before carbonation, the average carbon content of all AAM was approximately 0.02 g/mL, and the carbon contents of SL100, SL75, and SL50 increased to approximately 0.19 g/mL, 0.17 g/mL, and 0.13 g/mL, respectively, after carbonation. The result demonstrated the progress of CO_2_ binding (carbonation). In addition, the non-carbonated and carbonated areas of SL100, SL75 and SL50 were taken, respectively, for XRD analysis. The obtained diffractograms before and after carbonation are shown in Figure 7. It can be observed that the mass ratio of N-A-S-H increased from SL100 to SL50, which was reasonable considering the calcium content was lower when a higher amount of slag was replaced by the fly ash. The ongoing carbonation was verified by the generated peak for the quartz (marked with “Q” in Figure 7) after the carbonation of AAM, revealing the decalcification of calcium bearing materials. In addition, Figure 7 shows that the carbonation product was not only in the form of calcite; other forms of calcite carbonate, such as vaterite and aragonite, were also generated by AAM carbonation. The result agreed with previous research, where multiple forms of calcium carbonate were detected from naturally carbonated AAM [20,22]. The relative mass ratio of calcite, aragonite, and vaterite was calculated, as shown in Table 2. Note that no calibration substance was added in the XRD samples, meaning Table 2 only represents the relative mass ratio of the calcium carbonate to all the crystalline phases in tested sample. As shown, before carbonation, since the amorphous hydration product was not counted, the main crystalline phase was calcite, while after carbonation, since other calcium carbonate phases (e.g., vaterite, aragonite) popped up, the mass ratio of calcite decreased. The result revealed a transformation of an amorphous hydration product to the calcium carbonate, validating the ongoing carbonation. 

Combining phenolphthalein spray and XRD analysis, the ongoing carbonation and the reliability of CT scan were both confirmed in the present study. 

### 3.4. Spatial Distribution of Gray-Scale Value 

**Figure 8:**
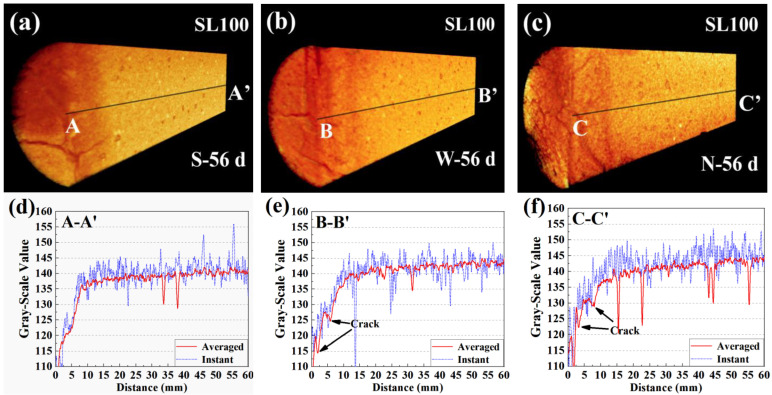
Spatial distribution of gray-scale value for SL100: “a”, “b”, and “c” stand for 3D distributions of gray-scale value of SL100 subjected to 0 m/s, 2.5 m/s, and 5.5 m/s windy environments, respectively; “d”, “e”, and “f” stand for 1D distributions of gray-scale value of SL100 subjected to 0 m/s, 2.5 m/s, and 5.5 m/s windy environments, respectively.

**Figure 9:**
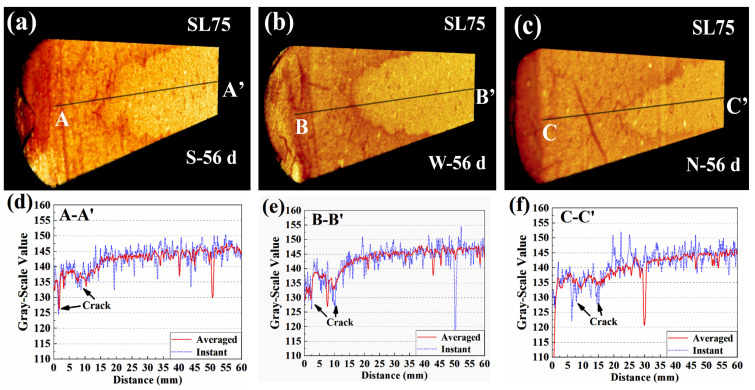
Spatial distribution of gray-scale value for SL75. “a”, “b”, and “c” stand for 3D distributions of gray-scale value of SL75 subjected to 0 m/s, 2.5 m/s, and 5.5 m/s windy environments, respectively; “d”, “e”, and “f” stand for 1D distributions of gray-scale value of SL75 subjected to 0 m/s, 2.5 m/s, and 5.5 m/s windy environments, respectively.

**Figure 10:**
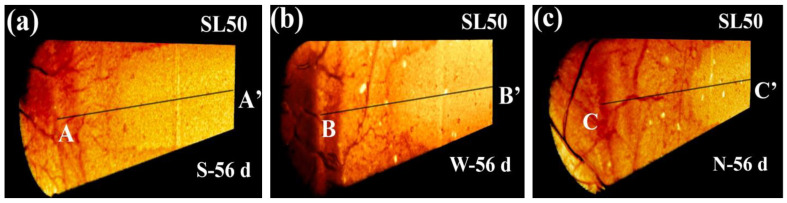

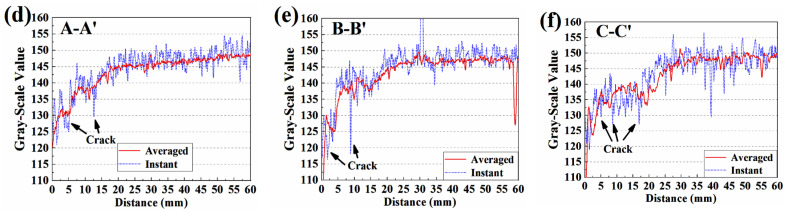
Spatial distribution of gray-scale value for SL50: “a”, “b”, and “c” stand for 3D distributions of gray-scale value of SL50 subjected to 0 m/s, 2.5 m/s, and 5.5 m/s windy environments, respectively; “d”, “e”, and “f” stand for 1D distributions of gray-scale value of SL50 subjected to 0 m/s, 2.5 m/s, and 5.5 m/s windy environments, respectively.

Each cross section was divided equally, and the series of arch were rendered to exhibit the cracked and carbonated areas of the carbonation face and on the axial plane. Moreover, linear scanning was performed along the axis of each cylindrical specimen, and 1D distribution of gray-scale value along the carbonation depth was measured. Both 3D rendering and 1D gray-scale value profiles are shown in Figure 8, Figure 9 and Figure 10. 

Figure 8a–c present the 3D renderings of gray-scale value for SL100. From Figure 8a–c, the specimens were exposed to environments with wind speeds of 0 m/s, 2.5 m/s, and 5.5 m/s, respectively. The 3D renderings are presented in the format of a temperature map, which means the higher the local gray-scale value, the brighter the area appears. Similar with Figure 3, the carbonated area appeared darker, which highlighted the reduced gray-scale value by carbonation. Moreover, crack networks were observable in the carbonated area, especially under the wind speed of 5.5 m/s. 

Figure 8d–f show the 1D distributions of gray-scale value along the carbonation depth. To ravel out the disturbance of the irregular-shaped carbonation front, both instant and averaged (averaged from 80 instant profiles) 1D gray-scale value profiles are presented. The gray-scale value of the non-carbonated area was in the range of 135 to 145, but that decreased drastically after carbonation. Based on the reduced gray-scale value, the carbonation depths of SL100 at 56 d of carbonation were estimated to be approximately 110 mm, 120 mm, and 150 mm, respectively, when exposed to 0 m/s, 2.5 m/s, and 5.5 m/s windy environments. The result highlighted the carbonation promoted by the wind. These things considered, judging from the averaged 1D profiles, the carbonation depths under all windy environments were quite similar. Even so, undulation was revealed around the carbonated part in the 2.5 m/s and 5.5 m/s profiles, which suggested ongoing of cracking. The whole carbonation process can be regarded as diffusion-controlling if a narrow carbonation front is revealed, while a broader carbonation front suggests that the investigated carbonation is not solely controlled by diffusion [9,41,42,43]. Detailed to the present study, unlike the observation earlier, the carbonation front at 56 d of carbonation was broader based on an instant 1D profile (especially for 5.5 m/s windy environment). The result suggested that the carbonation of SL100 could no longer be regarded as a diffusion-controlling progress, especially facing high windy environments. 

Figure 9 shows the 3D renderings and 1D gray-scale value profiles for SL75. The carbonation depths at 56 d were read to be approximately 180 mm, 200 mm, and 230 mm, respectively, when exposing to 0 m/s, 2.5 m/s, and 5.5 m/s environments. Obviously, the carbonation resistance of S75 was weaker than that of SL100, but the evolving pattern under windy environments was still compatible. For the averaged 1D gray-scale value profile, the carbonation front in SL75 was broad under all windy environments, which was mainly due to serious side carbonation. For the instant 1D profiles, all fronts were still broader than that in the literature [9,44], which served as compelling evidence that the carbonation of AAM could no longer be regarded as a diffusion-controlling progress when facing a windy environment. The reason for the altered carbonation mechanism is given here: random dispersed cracks pervaded the carbonated area of AAM, which changed the direction of CO_2_ diffusion (no longer perpendicular to the carbonation face), and the carbonation front shape was thus more irregular. In addition, compared to SL100, the extent of cracking was higher for SL75 (see the larger extent of fluctuation on SL75 profile), and the carbonation front was thus broader. To be strict, it should be noted here that the moisture released by carbonation could escape from the new-generated cracks, which also contributed to the fluctuation of 1D gray-scale value profile [44]. 

Figure 10 shows the 3D rendering and 1D gray-scale value profile of SL50. The carbonation depths of SL50 were read to be approximately 200 mm, 220 mm, and 250 mm, respectively, when exposed to 0 m/s, 2.5 m/s, and 5.5 m/s windy environments. Similar to SL100 and SL75, the carbonation speed was faster when facing environment of higher wind speed, and the cracking was more significant in SL50 (see the perforative cracks in the carbonated area). The carbonated front on the averaged 1D profile was narrower than those in SL75, which was mainly due to the less side carbonation in SL50. Quite the opposite, the instant 1D gray-scale value profile of SL50 fluctuated even more drastically, which highlighted a severe cracking in carbonated SL50. 

### 3.5. Carbonation under Variant Inner Humidity

**Figure 11:**
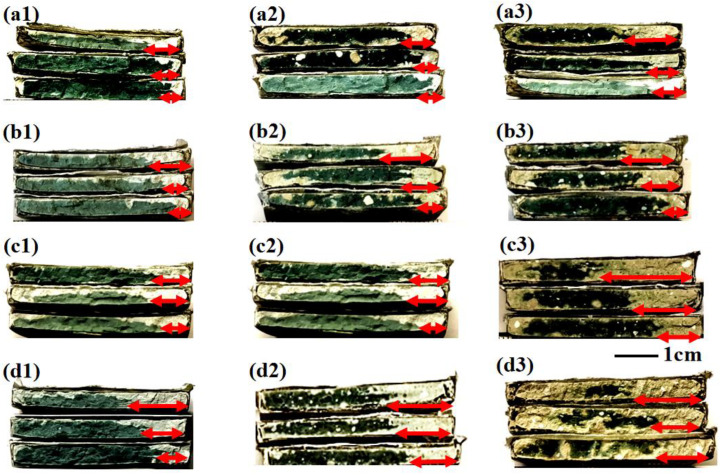
Typical cross sections of partly carbonated slices: (**a**–**c**), and (**d**) stand for 12 d, 28 d, 56 d and 112 d of carbonation, respectively; suffix “1”, “2”, and “3” stand for SL100, SL75, and SL50 respectively; from top to bottom, the slices were related to AAM preconditioned at 30%, 50%, and 70% RH.

Previous research based on the cylindrical specimens verified that the windy environment promoted carbonation. However, all above tests were based on specimens cured in a sealed condition, and the inner humidity of the tested specimen was thus unbalanced. In order to better understand the effect of wind on the evolved inner humidity, and further, on the carbonation behavior of AAM under windy environments, the inner humidity of AAM was balanced carefully before carbonation. The specimens after mass balancing were taken for carbonation under a 2.5 m/s windy environment. After 12 d, 28 d, 56 d, and 112 d of carbonation, the specimens were split, respectively, and the exposed cross sections are shown in Figure 11. In each sub-figure of Figure 11, the initial humidity before carbonation were 30%, 50%, and 70%, respectively, from the top to the bottom. 

Since slag was used as the raw material, the alkali-activated specimen was tinged with green in the non-carbonated state [42]. The area shifted to white after carbonation because of the decalcification of C-(A)-S-H, which could be used to trace the carbonation front. Apparently, the AAM of lower humidity carbonated faster, which seemed inconsistent with previous observation, where the carbonation speed attained its climax under an intermediate humidity level (40%~60%) [35]. In fact, a consensus has been attained already on the relationship between the humidity and the carbonation speed: neither excessively high (hinders the diffusion of gaseous CO_2_) nor excessively low humidity (lack of aqueous phase to support the chemical reaction) benefits carbonation, and rapid carbonation could be achieved only under an intermediate humidity level [45]. However, in the present study, the carbonation speed kept increasing when reducing the inner humidity. The different methods to control the humidity in the previous research and here was the main cause for the difference: in previous research, environmental humidity was designed to study the carbonation behavior under variant humidity, while in the present study, both the environmental humidity and the wind velocity were set as still, and the inner humidity was adjusted. Therefore, in the present study, lower humidity was more preferable, as it benefited the intrusion of both gaseous CO_2_ and moisture, and the carbonation speed was spontaneously faster. In addition, AAM instead of OPC was used in the present study, and the risk of cracking was therefore higher [21,23], especially when mass balanced under lower humidity. The new generated cracks under lower humidity could also promote the intrusion of moisture and CO_2_, leading to faster carbonation. 

**Figure 12:**
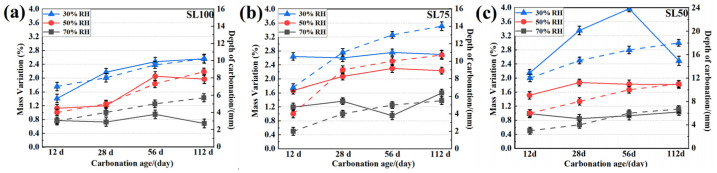
Mass Variation (solid lines)/carbonation depth (dashed lines) vs. carbonation age for (**a**) SL100, (**b**) SL75 and (**c**) SL50.

Compared among S100, S75, and S50, it is obvious that the carbonation speed was faster when a larger amount of fly ash was used to replace the slag, regardless of the inner humidity. Moreover, the side carbonation was also severer in fly ash-blended groups. Both results highlighted the vulnerability of fly ash-blended specimen, and its mix proportion should thus be carefully designed. To quantitatively illustrate the evolution of weight and carbonation depth along the carbonation age, both the mass variation and the measured carbonation depth are displayed in Figure 12. As shown, the mass of AAM samples increased more significantly in drier samples, which suggested water absorption. In addition, between 56 d and 112 d of carbonation, even though the carbonation depth still increased, the mass of SL50, SL75 and SL100 unanimously reduced, revealing the re-equilibrium of the inner humidity through the release of moisture. Finally, it should be noted here, the carbonation speed in Figure 12 was slower than that in Figure 3, Figure 4 and Figure 5. The slower carbonation in Figure 12 revealed better carbonation resistance in the sliced sample, which was most probably due to the continuous hydration during the mass balancing. 

In summary, the inner humidity of AAM did not evolve simultaneously with the outer atmosphere. Therefore, a windy environment, which promoted the exchange of humidity between the sample inside and the outer atmosphere, was more beneficial for carbonation. Furthermore, lower inner humidity favored the intrusion of both moisture and CO_2_, thus the carbonation resistance of AAM was weaker. Therefore, it is of high importance to understand the carbonation behavior under a windy environment, especially for alkali-activated concretes. 

## 4. Discussion

### 4.1. Method to Reveal the Carbonation-Incurred Cracking

Serving as the “fast path” to support CO_2_ diffusion, the presence of cracks would significantly attenuate the carbonation resistance of concrete structures [21,23]. Even so, previous investigations focused mainly on OPC, in which cracking resistance was better than that of AAM, and the promotion on the carbonation by cracking was not significant. However, AAM was used in the present study, which exhibited weaker cracking resistance, so the promotion on carbonation by cracking was non-ignorable. 

Vivid observation of the cracking during carbonation is never an easy task. For traditional methods such as scanning electron microscopy (SEM) [46] or mercury intrusion porosimetry (MIP) [47], even though their precision level are sufficient for the observation of cracks, the pretreatment is indispensable. Since new cracks may generate along the pretreatment, the observation of the cracking is less trustable. For the non-destructive method such as computed tomography (CT) [35], even though the technique renders the real microstructure of the tested sample, the observation on cracking is still difficult, as the resolution is inadequate (approximately 89 μm in the present study). 

**Figure 13:**
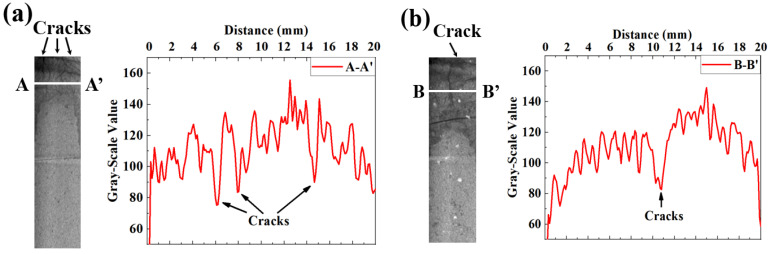
Identification of cracking through linear distribution of gray-scale value: (**a**,**b**) stand for SL50 carbonated under 0 m/s and 5.5 m/s windy environments, separately.

In order to reveal the cracking, the distribution of gray-scale value was revisited. Even though the tiny cracks were invisible due to CT’s limited resolution, the fluctuation of the local gray-scale value still “hinted” at the existence of cracking. Take SL50 at 56 d of carbonation as an example (see Figure 13, exposed, respectively, to 2.5 m/s and 5.5 m/s windy environments): even though the crack morphology was invisible, the anomalous low gray-scale value caused by both cracking and subsequent carbonation still indirectly pointed out the existence of cracking. 

Cracking can explain the occurrence of side carbonation, which describes the anomalous carbonation through the leaked side faces of the tested specimen. Since the cracks were more preferable to breed or propagate in the zones of lower strength, the interface between the side face and the sealing mold (PVC tube in the present study) was the most probable place to crack. Even so, as shown in the present study, if the cracking resistance of the AAM matrix was low, the lead of side carbonation would be less significant (see Figure 5 and Figure 10). Although it seems not an urgent issue to control the side carbonation, as it only occupies a few millimeters beneath the side face of concrete structures, the side carbonation of a sliced specimen should still be handled carefully if the inner humidity should be balanced ahead of carbonation (e.g., to study the effect of inner humidity on the carbonation), as it may disturb the designed 1D carbonation. 

### 4.2. Applicability of AAM under Windy Environment

The carbonation coefficient A ($\mathrm{mm}/\sqrt{d}$) was calculated via Equation (1) [48].

(1) $$D=D_{0}+A\times \surd t$$

where t stands for the carbonation age, and D and D_0_ stand separately for the carbonation depth at the carbonation age t and at 0 d. 

The carbonation coefficient was fitted according to Figure 12. The efficient of SL100, SL75, and SL50 were estimated as 0.2 $\mathrm{mm}/\sqrt{d}$ to 0.4 $\mathrm{mm}/\sqrt{d}$, 0.3 $\mathrm{mm}/\sqrt{d}$ to 0.7 $\mathrm{mm}/\sqrt{d}$, and 0.5 $\mathrm{mm}/\sqrt{d}$ to 0.6 $\mathrm{mm}/\sqrt{d},$ respectively. The variation of the carbonation coefficient mainly resulted from the variant initial humidity level, as the calculated carbonation coefficient would be relatively larger when facing a drier environment. These things considered, since current carbonation was performed under an accelerated condition, the obtained carbonation coefficient needed to be further transferred to comply with the natural environment. The transition was made according to Equation (2) [48].

(2) $$\frac{A_{a}}{A_{n}}=\sqrt{\frac{c_{a}}{c_{n}}}$$

where A_a_ and A_n_ stand separately for the carbonation depth under accelerated and natural condition, c_a_ and c_n_ stand separately for the CO_2_ concentration in laboratory and in the real circumstance. 

After the transition, the efficient of SL100, SL75, and SL50 were estimated as 0.035 $\mathrm{mm}/\sqrt{d}$ to 0.070 $\mathrm{mm}/\sqrt{d}$, 0.052 $\mathrm{mm}/\sqrt{d}$ to 0.121 $\mathrm{mm}/\sqrt{d}$, and 0.087 $\mathrm{mm}/\sqrt{d}$ to 0.104 $\mathrm{mm}/\sqrt{d}$, respectively, when facing a natural environment. Since the service life of most residential buildings in China was designed to be no less than 70 years, the carbonation depths at 70 years of carbonation were calculated [49]. The carbonation depths of SL100, SL75, and SL50 were about 8.59 mm to 15.19 mm, 18.78mm to 39.56 mm, and 15.90 mm to 28.62 mm, respectively, after 70 year of service. Considering that the concrete cover thickness was mostly designed as 30 mm according to the China standard GB T50082-2009 [49], current mix design was mostly acceptable for construction unless facing an extremely dry environment. 

The carbonation resistance of AAM in the present study was unusually better than that reported in the literature. According to Nedeljković et al. [50], the carbonation depth of AAM attained approximately 20 mm after 98 d of accelerated carbonation (1% CO_2_). Further, in previous work based on OPC, the carbonation depth attained 20 mm after 100 d of carbonation (3% CO_2_) [44]. The most probable reason for the enhanced carbonation resistance in this study was the lower water-to-binder ratio used (0.45), which guaranteed a refined microstructure, “cutting off” the path for CO_2_ diffusion. However, considering that such a low water-to-binder ratio was rarely used in real construction projects, the carbonation resistance of AAM, especially under a windy environment, should still be improved in the future. 

## 5. Conclusions and Future Work

### 5.1. Conclusions

In this study, a windy environment was simulated with the assistance of a self-developed wind tunnel, and alkali-activated slag/fly ash paste specimens were then carbonated under windy environments of variant velocity. To reveal the effect of inner humidity on carbonation, sliced alkali-activated materials (AAM) were mass-balanced as well to variant humidity, and the slices were carbonated under a 2.5 m/s windy environment. Based on computed tomography (CT), phenolphthalein spray, and X-ray diffraction (XRD), the microstructure at variant carbonation ages was rendered, and the effect of cracking and inner humidity on carbonation was systematically investigated. Several conclusions can be drawn from present study:

(1) Wind was capable of promoting the exchange of moisture between the sample inside and the outer atmosphere, leading to faster carbonation as compared to that under no wind environment.
(2) The gray-scale value of AAM was reduced by carbonation, mainly due to drastic cracking. In addition, the loss of water released during the decalcification of C-(A)-S-H also contributed to the reduction.
(3) The carbonation depths of SL100 at 56 d of carbonation were approximately 110 mm, 120 mm, and 150 mm, respectively, when exposed to 0 m/s, 2.5 m/s, and 5.5 m/s windy environments. Compared with OPC, the carbonation front on the 1D instant gray-scale value profile was broader in AAM, suggesting the progress was no longer under the sole control of diffusion.
(4) The carbonation depths of SL75 at 56 d were read to be approximately 180 mm, 200 mm, and 230 mm, respectively, when exposed to 0 m/s, 2.5 m/s, and 5.5 m/s environments. Furthermore, severe cracking was observed in the carbonated area, leading to a significant fluctuation in the carbonated area of instant 1D gray-scale value profile.
(5) The carbonation depths of SL50 at 56 d of carbonation were read to be approximately 200 mm, 220 mm, and 250 mm, respectively, when exposed to 0 m/s, 2.5 m/s, and 5.5 m/s environments. With weaker cracking resistance as compared to SL75, the side carbonation of SL50 was less significant than that in SL75.
(6) When preconditioned to lower inner humidity, the carbonation rate of AAM tended to be faster, as a larger gaseous space available to benefit both the intrusion of CO_2_ and moisture. Furthermore, severer cracking in AAM of lower inner humidity also contributed to the faster carbonation.

### 5.2. Future Work

In the present study, AAM was carbonated under variant windy environments. Although the effect of wind on AAM carbonation was verified, the present work was limited to pastes. Considering that the incorporation of fiber/aggregate can effectively restrict cracking, fiber reinforced mortars/concretes should be adopted in future to understand the carbonation behavior under windy environments. Furthermore, the present work was restricted to the leeward face, but much difference may be drawn when performing carbonation on the windward face, as the effect of wind pressure was involved as well. Therefore, the carbonation behavior on the windward face should be studied next. Moreover, no enhancing strategy to resist the AAM carbonation under windy environments was proposed in the present study, so new casting/preserving strategies should be developed in future to improve the concrete durability under the windy environment. All work mentioned above continues in our laboratory.

## Figures and Tables

**Table 1:** Chemical Composition of Cementitious Material (Mass Percentage,%).

Name	CaO	SiO_2_	Al_2_O_3_	MgO	P_2_O_5_	SO_3_	TiO_2_	Na_2_O	K_2_O	Fe_2_O_3_	Others
Slag	36.99	32.63	14.39	4.7	4.36	3.34	1.16	1.00	0.81	0.62	0
Fly ash	18	52	21	2.4	0.9	0.6	1.4	0.2	0.3	3.2	0

**Table 2:** Relative mass ratio of calcite, vaterite, and aragonite to the total crystalline phases (Mass Percentage,%).

Name	Calcite	Vaterite	Aragonite
SL100-C	10.66	62.76	6.37
SL100-NC	78.99	-	-
SL75-C	14.44	45.17	12.87
SL75-NC	83.44	-	-
SL50-C	36.59	8.12	30.68
SL50-NC	50.90	-	-

## Data Availability

Not applicable.
